# *BRCA1*-specific machine learning model predicts variant pathogenicity with high accuracy

**DOI:** 10.1152/physiolgenomics.00033.2023

**Published:** 2023-06-19

**Authors:** Mohannad Khandakji, Hind Hassan Ahmed Habish, Nawal Bakheet Salem Abdulla, Sitti Apsa Albani Kusasi, Nema Mahmoud Ghobashy Abdou, Hajer Mahmoud M. A. Al-Mulla, Reem Jawad A. A. Al Sulaiman, Salha M. Bu Jassoum, Borbala Mifsud

**Affiliations:** ^1^Division of Genomics and Translational Biomedicine, College of Health and Life Sciences, Hamad Bin Khalifa University, Doha, Qatar; ^2^Hamad Dental Center, Hamad Medical Corporation, Doha, Qatar; ^3^National Center for Cancer Care and Research, Hamad Medical Corporation, Doha, Qatar; ^4^William Harvey Research Institute, Queen Mary University of London, Charterhouse Square, London, United Kingdom

**Keywords:** BRCA2, breast cancer, in silico predictions, ovarian cancer, VUS

## Abstract

Identification of novel *BRCA1* variants outpaces their clinical annotation which highlights the importance of developing accurate computational methods for risk assessment. Therefore our aim was to develop a *BRCA1*-specific machine learning model to predict the pathogenicity of all types of *BRCA1* variants and to apply this model and our previous *BRCA2-*specific model to assess *BRCA* variants of uncertain significance (VUS) among Qatari patients with breast cancer. We developed an XGBoost model that utilizes variant information such as position frequency and consequence as well as prediction scores from numerous in silico tools. We trained and tested the model with *BRCA1* variants that were reviewed and classified by the Evidence-Based Network for the Interpretation of Germline Mutant Alleles (ENIGMA) consortium. In addition we tested the model’s performance on an independent set of missense variants of uncertain significance with experimentally determined functional scores. The model performed excellently in predicting the pathogenicity of ENIGMA-classified variants (accuracy: 99.9%) and in predicting the functional consequence of the independent set of missense variants (accuracy: 93.4%). Moreover it predicted 2 115 potentially pathogenic variants among the 31 058 unreviewed *BRCA1* variants in the *BRCA* exchange database. Using two *BRCA*-specific models we did not identify any pathogenic *BRCA1* variants among those found in patients in Qatar but predicted four potentially pathogenic *BRCA2* variants, which could be prioritized for functional validation.

## Graphical abstract

**Figure:**
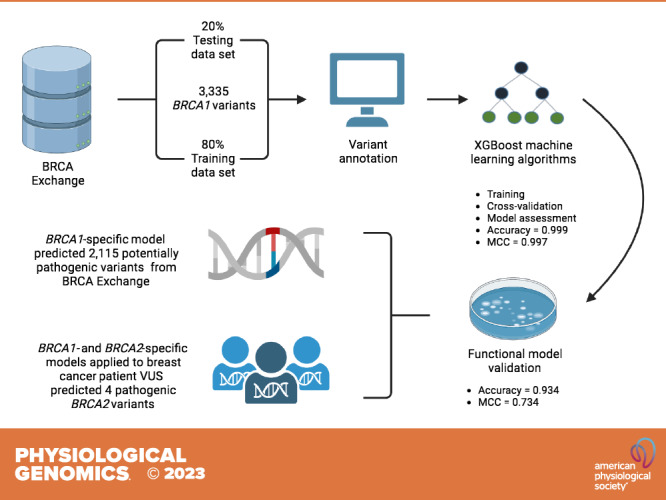

## INTRODUCTION

The *BRCA1* and *BRCA2* genes are tumor suppressor genes that encode for proteins involved in maintaining the integrity of the genetic material by repairing damaged DNA or destroying the cell if it is beyond repair (1, 2). When such genes are altered, cells can proliferate uncontrollably leading to cancer. BRCA proteins repair DNA double-strand breaks (DSBs) by the error-free homologous recombination (HR) and control DNA damage in cell cycle checkpoints (2). High penetrance *BRCA* mutations cause loss of the tumor suppressor function, which correlates with an increased risk of mainly breast and ovarian cancers (3). Harmful germline *BRCA* variants lead to Hereditary Breast and Ovarian Cancer syndrome (HBOC) that can affect both men and women. Affected individuals tend to develop cancer earlier in life and have a significantly greater risk of developing breast and/or ovarian cancer than the general population. In addition, although to a lesser degree, there is an increased risk of developing other types of cancer including prostate cancer, melanoma, pancreatic cancer, and others (4).

Deleterious *BRCA1* mutations are transmitted in an autosomal dominant pattern with cumulative cancer risk estimates for a 70-yr-old woman being between 40% and 87% for breast cancer and 16% to 68% for ovarian cancer (5–15). The likelihood of carrying an inherited mutation in *BRCA* varies across populations with an estimated prevalence in the general population between 0.2% and 0.8%; ∼1 in 400 to 139 individuals (16–19).

The Qatari population has a unique genetic profile characterized by a high consanguinity rate, large family size, and high prevalence of certain genetic disorders, mainly due to founder effects. According to the Qatar National Cancer Registry report (QNCR) of 2015, Qataris had a higher cumulative risk of getting cancer than other regional countries, and breast cancer was the most common cancer reported among women (20). In addition, HBOC is the most prevalent cancer syndrome identified in Qatar, accounting for ∼62% of all identified cancer syndromes, with *BRCA1* being the most commonly involved gene (21). It has also been observed that *BRCA1* mutations are highly prevalent among Qatari patients with breast cancer due to tribal founder effects that can partially explain the frequent young-onset diagnosis of breast cancer in the State of Qatar (22, 23).

Worldwide genome sequencing projects and genetic testing have uncovered thousands of variants in the *BRCA1* gene, many of which are variants of uncertain significance (VUS). Genetic screening of high-risk individuals at the National Center for Cancer Care and Research (NCCCR) has also identified 36 VUS in *BRCA1* and 54 VUS in *BRCA2*. *BRCA* VUS are particularly challenging for the management of families where they are identified, as they represent variants with undetermined risk of not only breast and ovarian cancers but also aggressive prostate and pancreatic cancers (24, 25). This highlights the importance of a consistent variant classification system with the use of genomics in patient care.

The American College of Medical Genetics and Genomics and the Association for Molecular Pathology created a joint standard of variant interpretation and classification (ACMG/AMP 2015 guidelines) (26). According to the ACMG/AMP guidelines, variant classification is based on the following information: population, computational prediction, functional, segregation, de novo, allelic, and other data (26). This approach in combination with the multifactorial likelihood-based model has been widely used, and it is the currently recognized method for classifying *BRCA* variants by the international Evidence-Based Network for the Interpretation of Germline Mutant Alleles consortium (ENIGMA) that specializes in clinical classification of *BRCA* variants (27–29). Nevertheless, there are still more than 31,000 *BRCA1* variants that have not yet been reviewed for classification. Moreover, as genetic data expand over time, the ACMG/AMP guidelines also indicate the need for an ongoing effort of VUS reclassifications by periodically reanalyzing these variants using more recent and updated data (26).

Numerous in silico prediction tools have been developed for assessing variants’ effect on the encoded proteins. The predictions from such algorithms are one of the eight evidence criteria required for variant interpretation by the ACMG/AMP guidelines. The guidelines state: “If all of the in silico programs tested agree on the prediction, then this evidence can be counted as supporting. If in silico predictions disagree, however, then this evidence should not be used in classifying a variant.” (18). Thus, using different combinations of in silico algorithms for variant interpretation can lead to discordant classifications. In fact, the assessment of the ACMG/AMP guidelines by the Clinical Sequence Exploratory Research consortium (CSER) revealed that the use of different combinations of in silico algorithms is a major source of inconsistencies in variant classifications across clinical laboratories and that the ACMG/AMP guideline for computational algorithm usage shall be aided by further recommendations (30).

The existing *BRCA1* gene-specific predictive model is based on a combination of different in silico prediction tools and is limited to missense variants in the critical protein domains that are known to be associated with impaired function (31). Moreover, it was based on small numbers of known damaging mutations (trained and tested with only 259 *BRCA1* damaging variants) and had a moderate Matthews Correlation Coefficient (MCC) of 0.66.

We aim to develop an improved and a more comprehensive *BRCA1* gene-specific pathogenicity prediction model for all types of variants to overcome the limitations of existing variant effect predictors. Compared with previous models, our model will utilize novel features and predict pathogenicity according to the comprehensive ACMG/AMP guidelines, as applied by the ENIGMA consortium, for all types of *BRCA1* variants. We will also use this new model to predict all 31,058 unclassified *BRCA1* variants and prioritize them according to their level of predicted pathogenicity. Finally, we aim to investigate the spectrum of *BRCA* VUS among Qatari patients with breast cancer and analyze them with novel methodologies to predict their pathogenicity and assess the possibility of classifying them into a more specific category.

## MATERIALS AND METHODS

### *BRCA1* Set of Variants and Annotation

We downloaded *BRCA1* variants from the BRCA Exchange database (https://brcaexchange.org/variants; accessed on September 7, 2022). These contained variants curated and classified by the international ENIGMA consortium. At the time of this study, there were 34,394 *BRCA1* variants, of which 3,335 variants were reviewed by the ENIGMA expert panel and had known effects of being pathogenic (2,228), likely benign (441), or benign (666). We utilized the Ensembl Variant Effect Predictor for the analysis and annotation of the different genomic variants (32). It provides access to information on the affected transcript, protein, and noncoding region, as well as frequency, phenotypes associated with the variant, and other annotations. In addition, it provides access to numerous in silico prediction algorithms including those that are present in the dbNSFP database (33).

The in silico prediction tools we included in the model were BayesDel_addAF, BayesDel_noAF, bStatistic, CADD, ClinPred, DANN, Eigen, EigenPC, FATHMM-XF coding, FATHMM-MKL coding, GenoCanyon, GERP++RS, GM12878fitCons, H1hESCfitCons, HUVECfitCons, integratedfitCons, LRT, MaxEntScan, MCAP, MetaLR, MetaSVM, MutationAssessor, MutationTaster2, MutPred, MPC, MVP, phastCons, PhyloP, Polyphen, PrimateAI, PROVEAN, REVEL, SIFT, SiPhy, SpliceAI, and VEST4. For those predictions, we used the rank scores whenever they were provided. The detailed list is presented in Supplemental Table S1. The other variables that were also collected from VEP included the position of the variant, number of bases involved based on reference and alternative alleles (“variant length”), variant association with phenotype, presence as a somatic mutation, variant impact, and variant consequence. As previously, the variant consequence variables were ranked based on the assumed pathogenicity of the effect with downstream variants having the least effect and stop-gained variants having the highest effect (34). We obtained the population frequency of the variants from both the BRCA Exchange database and from VEP. We used the highest frequency reported for any given variant as a variable called “maximum allele frequency” in the model.

### Model Building

We chose the XGBoost (Extreme Gradient Boosting) framework for building the gene-specific model because it implements a highly flexible, optimized, distributed gradient boosting machine learning algorithm (35). Moreover, when compared with other algorithms, it performed well in predicting the functional impact of *BRCA1* and *BRCA2* missense variants (31, 34). We also have recently applied an XGBoost model to predict the ENIGMA benign/pathogenic binary classification for *BRCA2* variants, and it outperformed all previous prediction models (34).

We used the XGBoost R package (version 1.4.1.1) with default parameters (booster = “gbtree,” objective = “binary:logistic”, eta = 0.3, gamma = 0, max_depth = 6, min_child_weight = 1, subsample = 1, colsample_bytree = 1, nrounds = 100) to train a classifier model on the variant annotations for predicting pathogenicity. The depth, nrounds, and eta hyperparameters impact the model’s performance and complexity. Depth determines the size of the decision tree. Higher depth allows the model to capture more complex relationships between the input features and the outcome, but increases the risk of overfitting. The nrounds parameter sets the number of iterations or trees that are used by the model. Higher nrounds can also lead to overfitting. The eta parameter controls the learning rate or step size used during the boosting process. A smaller eta value results in a slower, but potentially more accurate learning process, whereas a larger eta value results in a faster but potentially less accurate learning process. A small eta value is usually preferred, because it helps to prevent overfitting and improves the generalization of the model. Accordingly, we tested a combination of different hyperparameters; adjusting the depth (3, 4, 5, 6, and 12) and nrounds values (100, 200, and 300) did not change the performance of these models. Moreover, we tested different eta values (0.15, 0.3, and 0.6) and found that the performance was the best at the default setting of eta 0.3.

Pathogenicity was based on the ENIGMA expert panel’s review. Therefore, only the variants that have been reviewed were included in building the model (3,335 variants), and they were split into 80% training set and 20% test set. The reviewed variants included “benign,” “likely benign,” and “pathogenic” variants according to the ENIGMA classification criteria version 2.5.1 (https://enigmaconsortium.org/library/general-documents/enigma-classification-criteria/). The model was trained to predict the expert classification of either pathogenic or benign (“benign” and “likely benign”) variants and we performed five-fold cross validation of the model. The gain (xgb.plot.importance function) and the Shapely values (xgb.plot.shap function) were used to assess which are the most important features and most predictive characteristics. Finally, we predicted the pathogenicity of the 31,058 unreviewed *BRCA1* variants and the 36 *BRCA1* VUS found in the Qatari breast cancer patient cohort. The *BRCA2*-specific model (32) was applied to predict the effect of 54 *BRCA2* VUS from the same cohort.

### Testing the Model on Independent VUS with Functional Data

Bouwman et al. (36) assessed the functional effect of 238 *BRCA1* VUS on their ability to complement *Brca1*-deficient mouse embryonic stem cells in homologous recombination DNA repair (HRR), using cisplatin and olaparib sensitivity assays as well as direct repeat GFP (DR-GFP) HRR assay. Out of the 238 variants, 213 were not reviewed by the ENIGMA consortium and were not included in building the XGBoost model. Accordingly, those variants were used for independent assessment of the model on missense VUS. Out of the 213 variants, 27 showed functional defects according to cisplatin sensitivity assay alone, and 25 were deleterious in all three assays.

### The ROC Curve Analyses

The receiver operating characteristic (ROC) curve analyses were performed for each in silico prediction tool using Stata/SE statistical software (v. 11.1), and the area under the ROC curve was used to compare the different tools and to find the best cutoff points when needed. All statistical tests were two-tailed and *P* values less than 0.05 were considered statistically significant. It should be noted that the receiver operating characteristic (ROC) analysis of the different in silico predictions was performed using the full sample of 3,335 variants, and the AUCs were calculated for only those variants that had prediction scores for the given tool.

## RESULTS

### Variant Landscape

**Figure 1.**
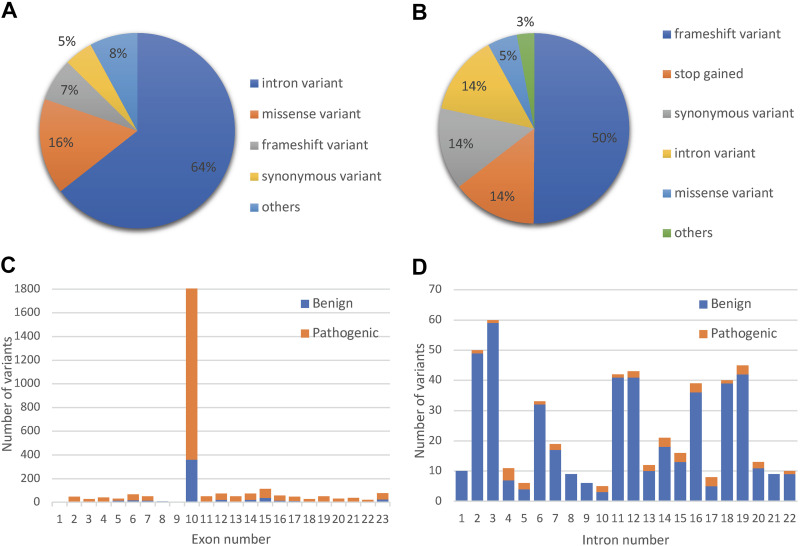
Distribution of consequences for *BRCA1* variants. *A*: consequences of all *BRCA1* variants. *B*: consequences of *BRCA1* variants that were classified by the ENIGMA expert panel and were used to build the models. *C*: the number of the reviewed pathogenic and benign variants per *BRCA1* exons. *D*: the number of the reviewed pathogenic and benign variants per *BRCA1* introns. ENIGMA, Evidence-Based Network for the Interpretation of Germline Mutant Alleles.

At the time of data collection, we downloaded 34,393 *BRCA1* variants from the BRCA exchange database; 11,021 were present along the 23 *BRCA1* exons and 23,372 were found across its 22 introns. The majority of those variants were intron variants (64%) followed by missense (16%), frameshift (7%), and synonymous (5%) variants (Fig. 1*A*). Only 3,335 variants were reviewed by the expert panel and had a known effect: 2,228 were pathogenic and 1,107 were benign (likely benign, or benign/little significance). The distribution of variant consequences in the reviewed portion greatly differed from the distribution in all variants. The largest proportion of the 3,335 reviewed variants was frameshift (50%) followed by stop gained (14%), synonymous (14%), and intron variants (14%) (Fig. 1*B*). The drop in the proportion of missense variants among the reviewed ones (5%) compared with the 16% of all coding variants indicates that most missense variants could not be classified to either pathogenic or benign categories.

In the 3,335 reviewed variants, the highest number of both benign and pathogenic variants was found in *exon 10*, the largest *BRCA1* exon, and they accounted for 54% of all reviewed variants (Fig. 1*C*). As expected, the highest number of specifically missense variants was also present in *exon 10* (58% of all missense variants). However, *exon 10* had only benign missense variants (Supplemental Fig. S1, *A* and *B*). The majority of the pathogenic missense variants (61%) were present in *exons 17*, *3*, *30*, and *4*, which are in the BRCT and RING domains. Out of the 507 reviewed intron variants (Fig. 1*D*), only 37 were pathogenic and included splice region, acceptor, and donor variants.

### Predicting Pathogenicity of the ENIGMA-Reviewed Variants

We used an extreme gradient boosting machine learning algorithm (XGBoost) to develop the *BRCA1* gene-specific prediction model. The model was built and tested on *BRCA1* variants with known ENIGMA expert-classified variants (3,335 variants). Those variants were divided into training set (80%) and test set (20%). The training set included 2,668 variants and it was trained to predict the expert classification of either pathogenic (1,794) or benign (874) variants. The test data included 434 pathogenic and 233 benign variants.

**Figure 2.**
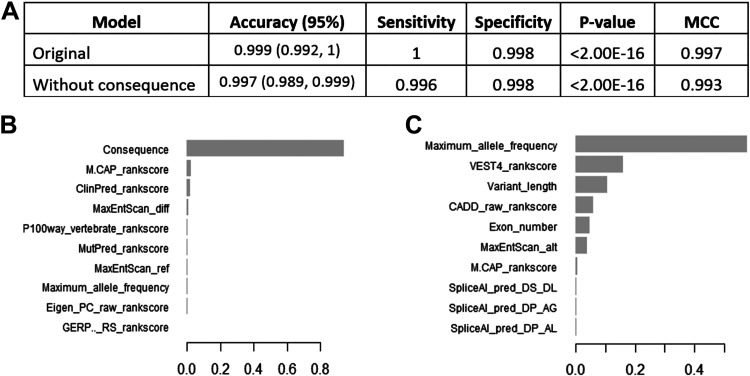
The *BRCA1* XGBoost models. *A*: the models’ characteristics. *B*: feature importance in the full XGBoost model. *C*: feature importance in the XGBoost model without consequence. AUC, area under the curve; MCC, Matthews correlation coefficient; XGBoost, extreme gradient boosting.

The model was used to predict the test group of 667 variants and yielded an accuracy of 0.999 and MCC of 0.997 with 100% sensitivity and 99.8% specificity (Fig. 2*A*). The most important feature was the variants’ type or “consequence” followed by a combination of different in silico tools’ rank scores (Fig. 2*B* and Supplemental Fig. S2*A*). Removing the consequence variable from the model did not affect the accuracy (0.997) and the maximum allele frequency became the most important feature, followed by the Variant Effect Scoring Tool (VEST4) rank score and the number of involved bases “variant length” (Fig. 2*C* and Supplemental Fig. S2*B*).

We also performed cross validation of the *BRCA1* model in five different subsamples that included random training and test groups. All models demonstrated similar accuracies between 99.7 and 99.9% (Supplemental Table S2). Similar to the original model, the variant consequence was the most important variable across the five subsamples, and when it was removed, the maximum allele frequency became the most important feature.

Next, we compared the performance of the XGBoost model with the performance of the in silico prediction tools, whose scores were used as input variables of the XGBoost model. The ROC analysis of the novel XGboost model demonstrated excellent diagnostic abilities with an area under the curve (AUC) equal to 99.8% in predicting all variants and an AUC of 1.00 in predicting missense variants (Supplemental Table S1). The different in silico prediction scores are only available for a subset of variants through VEP, which limits our abilities to compare them with our model. The MutPred rank score and the XGBoost model both had an AUC of 1.00. However, MutPred rank score was available for 57 variants only from VEP. We then applied MutPred to obtain a score for all 168 missense variants, and the ROC analysis of these yielded an AUC of 97.2%.

### Model Validation in Predicting VUS

We obtained VUS with functional data from a recent study by Bouwman et al. (36). After removing variants that were already included in building the model, 213 mostly missense VUS were used to validate the model (Supplemental Fig. S3*A*). Those VUS had functional data on their ability to complement *Brca1*-deficient mouse ES cells in homologous recombination DNA repair (HRR), using cisplatin and olaparib sensitivity assays and a direct repeat GFP HRR assay. Cisplatin sensitivity is most commonly used to assess *BRCA1* variant pathogenicity. Out of 213 VUS, 42 demonstrated functional defects in at least one assay, 25 of which were deleterious in all three assays.

**Figure 3.**
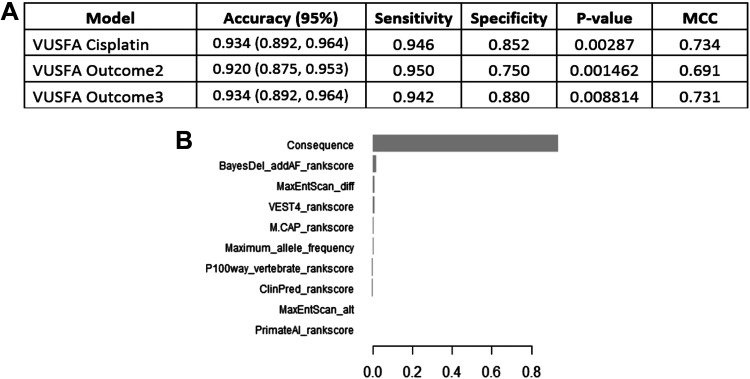
Using the *BRCA1* XGBoost model to predict VUS pathogenicity based on homologous recombination DNA repair (HRR) functional assay scores. *A*: the performance of the model on a set of pathogenic and benign variants according to different HRR assay outcomes: VUSFA Cisplatin is based on cisplatin sensitivity assay only, VUSFA Outcome2 requires at least two deleterious outcomes, and VUSFA Outcome3 requires deleterious results for all three assays for a variant to be considered pathogenic. *B*: feature importance of the XGBoost model trained on the whole reviewed dataset. AUC, area under the curve; MCC, Matthews correlation coefficient; VUS, variants of uncertain significance; XGBoost, extreme gradient boosting.

The *BRCA1* model was trained on the full set of the *BRCA1* variants (3,335), and it was used to predict the VUS that demonstrated functional defects. We tested the model in predicting the functional impact of three different pathogenic outcomes; the first outcome is based on cisplatin sensitivity assay only, in the second, pathogenic classification requires at least two deleterious outcomes, and the third outcome requires deleterious results for all three assays. The model demonstrated excellent diagnostic abilities for the three outcomes with accuracies between 0.92 and 0.93, sensitivity up to 0.95, and specificity up to 0.88. (Fig. 3*A*). Assessment of feature importance demonstrated that the most important variable was the variant consequence followed by the in silico prediction tool BayesDel addAF rank score (Fig. 3*B* and Supplemental Fig. S3*B*).

### Model Pathogenicity Predictions for the Unreviewed Variants

**Figure 4.**
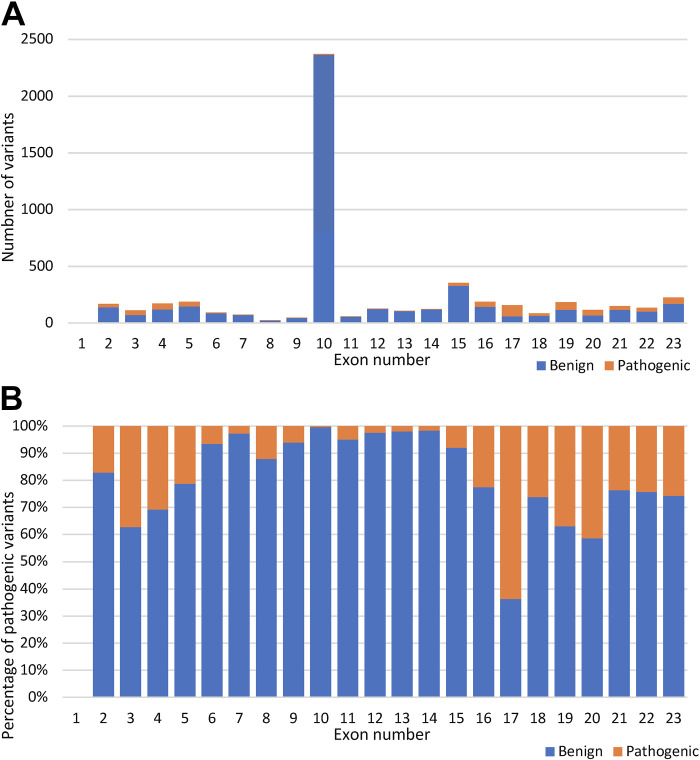
Comparison between the predicted pathogenic and benign variants across the *BRCA1* exons. *A*: the number of predicted pathogenic and benign missense variants (5,280) per *BRCA1* exons. *B*: percent distribution of predicted pathogenic and benign missense variants across the *BRCA1* exons.

Next, we used the gene-specific *BRCA1* model to predict the remaining 31,058 variants present in the BRCA Exchange database that are not yet reviewed by the expert panel. We predicted 2,115 variants to be pathogenic and prioritized them according to the total SHAP values of the different predictors (Supplemental Table S3). We predicted 635 pathogenic missense variants (Fig. 4*A*). The majority of those are either in the N-terminal RING domain (*exons 2–5*) or the C-terminal BRCT domain (exons 15–23); however, 33 were outside of those two domains, 9 of which were in exon 10. Exon 10 demonstrated the lowest rate of missense variant pathogenicity of 3.7% compared with 63% in exon 17, which had the highest rate (Fig. 4*B*).

### Pathogenicity Prediction of Qatari VUS

The Qatari National Center for Cancer Care and Research (NCCCR) genetic screening program uncovered 90 VUS in 78 patients with breast cancer; 29 patients with 36 *BRCA1* VUS and 49 patients with 54 *BRCA2* VUS.

Out of the 36 *BRCA1* VUS, 20 were not yet reviewed by the ENIGMA consortium and 3 were not present in the BRCA exchange database: 2 intron variants (c.4675 + 58 T > C and c.135-18T > C), and a 3-UTR variant (*1298 G > C) (Table 1). The remaining 12 variants were already classified by ENIGMA. We utilized the *BRCA1* model to predict the unreviewed and new variants, which classified all of them as benign variants.

**Table 1.** BRCA1 and BRCA2 variants of uncertain significance present in Qatari patients with breast cancer

Patient ID	VUS	Exon	BRCA Exchange	Consequence	Max Allele Frequency
*BRCA1*					
V006	c.3645C>G	10	Not yet reviewed	Missense variant	0.0000895
V0010	c.4675 + 58 T>C		Not present	Intron variant	
V0011	c.503A>C	7	Not yet reviewed	Missense variant	
	c.765G>A	10	Not yet reviewed	Synonymous variant	0.0038
	*655G>A	23	Not yet reviewed	3UTR variant	0.001087
	*1298G>C	23	Not present	3UTR variant	
	*1323A>G	23	Not yet reviewed	3UTR variant	0.0038
V0020	c.2176C>T	10	Not yet reviewed	Missense variant	0.00002
V0024	c.*1191A>G	23	Not yet reviewed	3 Prime UTR variant	
V0027	c.-264T>G		Not yet reviewed	Upstream gene variant	0.0004139
V0029	c.878C>T	10	Not yet reviewed	Missense variant	
V0037	c.2668G>A	10	Not yet reviewed	Missense variant	
V0042	c.1745C>T	10	Not yet reviewed	Missense variant	4.75828E-05
V0048	c.3587C>T	10	Not yet reviewed	Missense variant	0.00406504
V0058	c.994C>T	10	Not yet reviewed	Missense variant	0.000213311
V0059	c.3587C>T	10	Not yet reviewed	Missense variant	0.00406504
V0060	c.851A>G	10	Not yet reviewed	Missense variant	
V0077	c.2831G>A	10	Not yet reviewed	Missense variant	0.00006095
V0078	c.3555G>T	10	Not yet reviewed	Missense variant	
V0081	c.3555G>T	10	Not yet reviewed	Missense variant	
V0084	c.4636G>C	14	Not yet reviewed	Missense variant	0.000163806
V0085	c.135-18T>C		Not present	Intron variant	
V0087	c.3995G>T	10	Not yet reviewed	Missense variant	0.000208333
V0091	c.1405G>A	10	Not yet reviewed	Missense variant	
*BRCA2*					
V0014	c.2534C>T	11	Not yet reviewed	Missense variant	0.000167
V0012	c.6568G>A	11	Not yet reviewed	Missense variant	0.000066
V007	c.9839C>A	27	Not yet reviewed	Missense variant	0.0004946
V005	c.10062_10070delTGGTTCAAC	27	Not reported	Inframe deletion	
V0021	c.1627C>A	10	Not yet reviewed	Missense variant	
V0028	c.8411C>T	19	Not yet reviewed	Missense variant	
V0030	c.8687G>A	21	Not yet reviewed	Missense variant	0.000266667
V0031	c.8141A>G	18	Not yet reviewed	Missense variant	
V0034	c.516 + 18 T>C		Not yet reviewed	Intron variant	0.006
V0036	c.1478C>T	10	Not yet reviewed	Missense variant	
V0037	c.6761T>A	11	Not yet reviewed	Missense variant	
V0040	c.1550A>G	10	Not yet reviewed	Missense variant	0.000474834
V0041	c.353G>T	4	Not yet reviewed	Missense variant	
V0042	c.6827C>A	11	Not yet reviewed	Missense variant	0.0003048
V0043	c.1607C>A	10	Not yet reviewed	Missense variant	
V0044	c.5808G>A	11	Not yet reviewed	Missense variant	0.0001774
V0046	c.9562G>A	26	Not yet reviewed	Missense variant	
V0049	c.1826A>G	10	Not yet reviewed	Missense variant	0.000411523
V0051	c.280C>T	3	Not yet reviewed	Missense variant	0.000426439
V0053	c.2651C>T	11	Not yet reviewed	Missense variant	0.0003117
V0057	c.8774A>G	22	Not yet reviewed	Missense variant	8.35701E-05
V0061	c.5269T>C	11	Not yet reviewed	Missense variant	
V0062	c.4252A>G	11	Not yet reviewed	Missense variant	0.0008
V0064	c.1627C<A	10	Not yet reviewed	Missense variant	
V0066	c.5557_5558delTGinsCT	11	Not yet reviewed	Missense variant	
V0068	c.9754_9765del	27	Not present	Inframe deletion	
V0070	c.3983G>A	11	Not yet reviewed	Missense variant	0.0001843
V0071	c.280C>T	3	Not yet reviewed	Missense variant	0.000426439
V0072	c.8141A>G	18	Not yet reviewed	Missense variant	
V0074	c.8577A>C	20	Not yet reviewed	Missense variant	
V0076	c.9562G>A	26	Not yet reviewed	Missense variant	
V0082	c.4201G>A	11	Not yet reviewed	Missense variant	
V0083	c.8201C>G	18	Not present	Missense variant	
V0084	c.8488-5T>C		Not yet reviewed	Splice region variant	0.0061
V0088	c.5414A>G	11	Not yet reviewed	Missense variant	0.000370553
V0093	c.3904A>G	11	Not yet reviewed	Missense variant	0.000015647
V0096	c.7954G>A	17	Not yet reviewed	Missense variant	3.27654E-05
V0097	c.4090A>G	11	Not yet reviewed	Missense variant	4.02739E-05
V0098	c.9378G>C	25	Not yet reviewed	Missense variant	

Not yet reviewed, found in the BRCA exchange database but has not been reviewed; not present, not found in the BRCA exchange database; not reported, not found in any variant repositories. Variants predicted to be pathogenic are underlined. VUS, variants of uncertain significance.

Regarding the 54 *BRCA2* variants, 36 of them were not yet reviewed by the ENIGMA consortium, and 3 were not present in the BRCA exchange database: a missense variant (c.8201C > G) and 2 in-frame deletion variants (c.10062_10070del and c.9754_9765del). We applied our previously published *BRCA2-*specific model to classify the 39 *BRCA2* VUS. The model predicted four of them as pathogenic variants (Table 1): an in-frame deletion (c.9754_9765del), and three missense variants (c.5557_5558delTGinsCT, c.7954G > A, and c.9378G > C).

## DISCUSSION

The human *BRCA1* gene contains 23 coding exons. The largest is *exon 10.* Accordingly, it harbors the highest number of both benign and pathogenic variants. However, despite it being around 60% of the *BRCA1* coding sequence, *exon 10* had no confirmed pathogenic missense variants. That is why *exon 10* was referred to as a “coldspot,” or a region of the gene that is tolerant of variation and where pathogenic missense variants are unlikely. Such coldspots are consistent with the ACMG/AMP criteria for “strong benign” evidence, allowing them to be initially classified as likely benign in most instances (37). Interestingly, we predicted nine pathogenic missense variants in *exon 10*, seven of which were predicted to substantially decrease protein stability using I-Mutant 3.0 (38). Nevertheless, when the pathogenicity rate of missense variants was calculated for each exon alone, *exon 10* had the lowest rate of pathogenicity; only 3.7% of the missense variants in *exon 10* were pathogenic. The highest pathogenicity rate was observed in *exon 17* (63%), which is expected as exon 17 falls in the BRCT domain, one of the two BRCA1 critical domains.

Our XGBoost model accurately predicted the ENIGMA consortium’s final classification and demonstrated better diagnostic probabilities than the previous *BRCA1* model and other in silico prediction tools (31, 39). It is applicable not only to missense variants that are tested in functional assays but to all possible variant types.

The previous *BRCA1* model developed by Hart et al. (31) was limited to missense mutations in the RING and BRCT domains of the BRCA1 protein, which are known to be associated with impaired function; therefore, it is not known if variants in other domains that are predicted to cause damaging mutations are able to inhibit DNA repair. In addition, the existing BRCA1 model was trained and tested with only 263 pathogenic *BRCA1* variants. Being based on small numbers of known damaging mutations limits the models’ ability to capture the variability of variant data; thus, that model could not capture all the features of damaging variants. Finally, the previous model was based solely on different in silico prediction tools and did not include other variables that can support variant classification. Direct comparison of the performance of the model by Hart et al. (31) and our model is not possible due to the different sets and types of variants predicted, but we could examine the performance of the XGBoost model on missense variants in the test group alone and calculated the MCC. Our model had an MCC of 1 in comparison with the MCC of 0.66 reported for the previous *BRCA1* model. Moreover, the model had an MCC value of 0.997 in predicting the ENIGMA classifications for all types of variants, and an MCC of 0.934 in predicting the functional impact of a different set of missense variants as tested by homologous recombination DNA repair assays.

We did not optimize the model parameters; therefore, the model might perform better with optimal settings. Nevertheless, we did test the model with a combination of different depth (3, 4, 5, 6, and 12), nrounds (100, 200, and 300), and eta values (0.15, 0.3, and 0.3); however, we did not observe any improvements, which suggests that the default settings achieve the best performance.

The assessment of the ACMG/AMP guidelines by the CSER consortium revealed that the use of different combinations of in silico algorithms is a major source of inconsistencies in variant classification across clinical laboratories (30). Recent studies that aimed to reinterpret variants of uncertain significance in the *BRCA1* and *BRCA2* genes selected different prediction tools and set different criteria for defining supporting evidence of pathogenicity or benign impact, which can lead to different conclusions of variants (40, 41). The *BRCA1*-specific model is an accurate method for predicting the ENIGMA consortium classified pathogenicity of all types of variants in the *BRCA1* gene. Thereby, it can eliminate the classification inconsistencies that are inherent to using different prediction tools. Furthermore, we predicted the pathogenicity of 31,058 *BRCA1* variants that have not been classified by the ENIGMA consortium and prioritized them according to the predicted level of pathogenicity. This prioritized list can aid in the interpretation of *BRCA1* variants and guide future studies.

Finally, we applied this *BRCA1-*specific and the previously published *BRCA2*-specific models to predict the pathogenicity of variants of uncertain significance that were identified in patients with breast cancer by the Qatari National Center of Cancer Care and Research. We predicted four *BRCA2* VUS to be pathogenic: a missense variant (c.5557_5558delTGinsCT) at the BRCT repeat number 6 (BRC6) in *exon 11*, which is part of the RAD51-binding domain; a missense variant (c.7954G > A) at the helical part of the DNA binding domain in *exon 17*; a missense variant (c.9378G > C) at the third oligonucleotide/oligosaccharide-binding fold (OB3) of the DNA binding domain in *exon 25*; and an in-frame deletion (c.9754_9765del) at amino acids 3252–3255, which is close to the putative nuclear localization signals (NLS1: aa. 3263–3269) that are present at the second RAD51-binding domain (aa. 3270–3305) in *exon 27*. Further functional analysis of these variants will be required to confirm their pathogenicity.

## DATA AVAILABILITY

Variants used were downloaded from the BRCA Exchange database: https://brcaexchange.org/variants. A training and test data set and the script to create the machine learning model are available through GitHub: https://github.com/borimifsud/BRCA1_pathogenicity.

## SUPPLEMENTAL DATA

10.5281/zenodo.7840851 Supplemental Tables S1–S3 and Supplemental Figs. S1–S3: https://doi.org/10.5281/zenodo.7840851.

## GRANTS

This research was funded by the internal departmental funding at Hamad Bin Khalifa University.

## DISCLOSURES

No conflicts of interest, financial or otherwise, are declared by the authors.

## AUTHOR CONTRIBUTIONS

M.K. and B.M. conceived and designed research; M.K. and B.M. performed experiments; M.K., H.H.A.H., N.B.S.A., S.A.A.K., N.M.G.A., H.M.M.A.A.-M., R.J.A.A.A.S., S.M.B.J., and B.M. analyzed data; M.K. and B.M. interpreted results of experiments; M.K. prepared figures; M.K. drafted manuscript; M.K. and B.M. edited and revised manuscript; M.K., H.H.A.H., N.B.S.A., S.A.A.K., N.M.G.A., H.M.M.A.A.-M., R.J.A.A.A.S, S.M.B.J., and B.M. approved final version of manuscript.
